# Development of a School-Based Physical Activity Intervention Using an Integrated Approach: Project SMART

**DOI:** 10.3389/fpsyg.2021.648625

**Published:** 2021-08-13

**Authors:** Yeonhak Jung, Sheri L. Burson, Christine Julien, Dylan F. Bray, Darla M. Castelli

**Affiliations:** ^1^Department of Kinesiology, California State University, Northridge, Northridge, CA, United States; ^2^Department of Kinesiology and Health Education, The University of Texas at Austin, Austin, TX, United States; ^3^Department of Electrical and Computer Engineering, The University of Texas at Austin, Austin, TX, United States

**Keywords:** physical activity, community-based participatory research, self-determination theory, intervention mapping protocol, physical activity promotion, school-based physical activity

## Abstract

Physical activity (PA) is a health-protective factor with multiple benefits for school-age children, yet only 22% of children and adolescents living in the United States (United States) accrue the recommended amount of moderate to vigorous PA. Given the prevalence of insufficient PA among children, promoting and providing PA opportunities during the school day, especially when integrated into the curriculum and linked to the learning standards, is essential for children. The purpose of this paper is to describe the procedure for the development of a school-based PA program using an integrated approach through the modified intervention mapping protocol (IMP). A total of 22 physical education teachers and 167 children from five different elementary schools were involved in the process. The procedure includes the Self-Determination Theory (SDT) that provides a theoretical framework that plays a vital role in motivating students to have a physically active lifestyle. This study applied SDT and IMP to develop and pilot a PA intervention called Project SMART using an integrative community participatory approach. As a pilot PA intervention, Project SMART is an online educational game where the students navigate a virtual journey across the United States A class’s aggregate PA propels the students on their journey, where standards-based modules are unlocked to achieve STEM (science, technology, engineering, and math) and social-emotional learning outcomes while gaining an understanding of the importance of health behaviors and opportunities to habitually engage in healthy decision-making with the support of their peers. Although initially labor intensive for the researchers, the process of tailoring the intervention to the children’s contextual and cultural needs has implications for all theoretically grounded and evidence-based PA interventions.

## Introduction

Globally, the prevalence of childhood obesity and physical inactivity continues to rise (Guthold et al., 2020), as the daily recommendation of 60 min of moderate to vigorous physical activity (MVPA) each day is not met by 80% of all children [World Health Organization (WHO), 2019]. Survey data suggests that only 22% of children and adolescents living in the United States (United States) participate in the recommended amount of MVPA [Child and Adolescent Health Measurement Initiative (CAHMI), 2019]. Theoretically, schools are ideal for promoting PA, not only because children spend more than 6 h in schools each weekday but also because a whole-of-school approach engages members of the community like teachers, parents, and peers (Murillo Pardo et al., 2013; Mannocci et al., 2020). In addition, physical education in schools has been shown to have positive effects on children’s MVPA level (Lonsdale et al., 2013) and motivational processes to be active (Sevil-Serrano et al., 2020). Despite the potential for schools to integrate comprehensive models to provide PA opportunities for children in and around schools, such a shift to health-enhancing structures remains a work in progress, as evidence of school intervention effectiveness and impact on health outcomes is mixed (Langford et al., 2014; Love et al., 2019). Several existing systematic reviews and meta-analyses have examined the effectiveness of school-based interventions promoting PA only to find a small or non-significant impact on intervention compared to non-intervention participants (Dobbins et al., 2013; Jones et al., 2020; Kennedy et al., 2021). The primary concern is that many interventions are unresponsive to children’s needs in a given environment because they fail to integrate equity-centered and inclusive approaches (Golden and Earp, 2012; Breny, 2020).

Children have a range of PA experiences and opportunities, yet all too often, there are reports of health disparities among children residing in minority-majority communities and school districts (Gordon-Larsen et al., 2006; Ogden et al., 2015; Barbosa Filho et al., 2016). The *Healthy People 2030* report implicated disparities as a primary reason for adverse child and adolescent health outcomes [Office of Disease Prevention and Health Promotion (ODPHP), 2019]. Ethnic minority groups are less physically active and have higher obesity rates than children who meet the PA guidelines (Chang, 2019; Pfledderer et al., 2021). The risk for obesity is likely due to unhealthy eating and low accessibility to safe PA spaces, such as playgrounds, sidewalks, and recreational facilities. There is a paucity of research investigating how school PA interventions influence the PA of ethnic minorities and socioeconomically disadvantaged children and adolescents (Basch, 2011; Brusseau et al., 2016), as mistrust and a lack of health-first approaches inhibit access to this interest group. Additionally, cultural and economic characteristics influence the magnitude of health inequities (Cohen et al., 2017). Reduced PA participation and opportunities for engagement increase the odds of physical, mental, and cognitive health issues (Eime et al., 2013; Biddle et al., 2019). Thus, school PA interventions must be contextually and culturally grounded to impact children’s health and well-being.

Comprehensive School Physical Activity Programs (CSPAP) have the potential to reverse obesogenic trends by engaging multiple partners through five points of intervention: (a) physical education, (b) during the school day PA (e.g., recess, classroom PA), (c) staff involvement, (d) before- and after-school PA, and (e) community engagement (Ploeg et al., 2014; Burns et al., 2015; Pulling Kuhn et al., 2021). Given the multiplicity of models like CSPAP, there are natural barriers to their integration (Bates et al., 2020). School staff members, teachers, administrators, and community members are essential for providing PA opportunities but may not be prepared to do so; for example, a lack of teacher training, staff motivation, or school climate could be the main barriers to promoting PA (Herlitz et al., 2020). The school-university model (Patton, 2012; Castelli et al., 2013) is the traditional structure of partnerships to support health models like CSPAP, but many schools lack the readiness to implement such models (Phelps et al., 2019). The advantages of such partnerships include shared leadership, integration of knowledge, and relationship building, but the sustainability of a partnership is predicated upon the commitment and continued involvement of a champion facilitator, and the importance of these likely outweigh the other barriers (Parker et al., 2012). If successful, school-university collaborations address the potential shortcomings in teacher education or inexperience. The bidirectional relationships between schools and universities are well-intended, but few studies have examined the effects of school-based PA interventions on behavior using an integrative community-based participatory approach (Hogan et al., 2014; Wallace, 2019; Pulling Kuhn et al., 2021). Further, despite community engagement being included in the CSPAP, there is a paucity of research related to the role of community organizations in the promotion of PA. Accordingly, we sought to engage a university, community stakeholders, and one school district as co-investigators in intervention mapping to design a school-based PA intervention for elementary school children.

Project SMART is an outgrowth of The University of Texas at Austin Whole Communities-Whole Health (WCWH) VP for Research Grand Challenge, an equity-centered and community-first project focused on the longitudinal study of children’s health to improve the lives vulnerable families to address health inequities (see ^1^). WCWH brings together academic researchers from multiple disciplines, school personnel, community partners, and parents to co-design the longitudinal study. When community focus groups revealed collective concerns about obesity and physical inactivity, Project SMART was born. Modeled after KidsGoGreen (Gerosa et al., 2015; CLIMB, 2019), a game developed by another research group whose purpose was to change a family’s sustainable mobility habits, Project SMART was intended to be both educational and health-enhancing. Project SMART’s basic principle was to leverage the community relationships and learnings from the WCWH initiative to develop an online, cooperative game designed to use student PA as propulsion to travel across the virtual United States while unlocking learning modules aligned with educational standards. Secondarily, the long-term goal was to shift school cultures to develop health-first policies and shared responsibility for creating PA opportunities and participation for children and their families.

We expanded the ongoing interactions through WCWH to engage key stakeholders and care providers in a modified intervention mapping protocol (IMP; Bartholomew-Eldredge et al., 2016). Although this will be described in detail later, the IMP framework has utility and attempts to address the shortcomings of previous interventions that have only met a portion of students’ needs; based on these experiences, specific IMP steps for intervention design and development were used to ensure that the resulting intervention was contextually, culturally, and theoretically grounded.

This paper aims to describe the stages of the development of Project SMART using IMP in the form of a process paper. Process evaluations are vital in program implementation to ensure the program is implemented as intended, assess accessibility and acceptance among the participants, and identify issues early enough to make necessary adjustments [Centers for Diseases Control and Prevention (CDC), 2012]. The modified IMP steps were applied to develop the intervention because the fifth and sixth steps of the original IMP were further than the range of the present study. In addition to IMP, the Self-Determination Theory (SDT) has been found suitable for developing school-based intervention and promoting children’s PA (Ryan and Deci, 2000). The theoretical framework was found during the process of IMP, not chosen ahead of time. Therefore, as an integrated approach for this protocol, all processes will be described in the method section. Beyond the scope of this paper, the overall project aims are aligned threefold:

1. To assess the efficacy of Project SMART for increasing PA in 4th and 5th grade in schools serving primarily minority and socioeconomically disadvantaged communities.
2. To examine the mediation effects of Project SMART on children’s self-determination for PA, academic engagement, and academic performance, our secondary outcomes of interest.
3. To estimate the effects of dosage, fidelity, and gameplay elements on intervention effects on primary and secondary outcomes.

We hypothesized that the intervention would increase total weekly PA more than traditional instruction or intervention, aligned with the student needs, local context, and educational outcomes. Further, it was anticipated that relatedness, autonomy, and competence would mediate the Project SMART intervention’s effects. The effects of dosage, fidelity, and gameplay on the primary and secondary outcomes were mainly unknown. Children in 4th and 5th graders are approaching a developmental transition, which is when PA is reduced through dropout, especially among students not selected for sport participation. Only 25% of youth under the age of 6–17 years are currently meeting the PA guidelines (Piercy et al., 2018). Therefore, 4th and 5th graders were chosen as the population of interest. The ultimate goal is to conduct a cluster randomized wait-list control trial with 4th and 5th graders across multiple school districts, including minority and socioeconomically disadvantaged communities in Central Texas.

## Materials and Methods

Whole Communities-Whole Health initiative is a transdisciplinary, team science project initially funded by The University of Texas at Austin to fund the infrastructure for a longitudinal study of children’s health. Faculty from 12 different academic units, research staff, community strategy team (CST), community stakeholders, school personnel, and research staff represented the community of interest, and the disciplines of medicine, psychology, health communications, nursing, biology, and education are represented on the team. Using an integrated participatory approach, teachers, administrators, faculty, students, and community partners first built relationships. Second, the team shared resources for co-planned events and finally co-designed the pilot and longitudinal research study. Across 2 years, the WCWH research team co-hosted 52 different community events. The events included wellness fairs, town halls, parent and teacher focus groups, stakeholder meetings, and CST meetings. The CST was made up of 12 members who served as an advisory board for the large-scale project. Through the events, the research team became aware of the community’s concern for child obesity and PA. When asking teachers about their thoughts on implementing a PA program in their school or classroom, they identified the importance of the program also providing educational content that aligns with the Texas Essential Knowledge and Skills (TEKS,^2^). Further, teachers expressed concerns about competition between students leading to exclusion and other social and emotional issues. These qualitative data aided in the development of Project SMART, which was to address child PA while incorporating educational standards and require peer cooperation rather than students playing individually or competitively.

The Project SMART research effort was originally introduced by Julien et al. (2021) as an online cooperative game designed to increase PA in elementary students while teaching academic content driven by state standards. While this previous work described the technical efforts to construct the game and pilot deployment of technical feasibility in a small setting, this paper instead focuses on how the entire Project SMART was designed, developed, and applied in the school settings to increase PA among elementary students using IMP. The IMP steps served as the framework for undertaking the outlined methodologies used to ensure the PA intervention was systemically grounded in theory and guided by academic outcomes. This protocol guides the design of multi-level health promotion interventions and implementation strategies. Traditionally, the IMP consists of six steps: (a) defining the problem, (b) identifying outcomes and objectives, (c) selecting theory-based methods and practical strategies, (d) developing a program plan, (e) implementing the intervention, and (f) evaluating the program (Bartholomew-Eldredge et al., 2016). Although completing all six steps serves as a guideline for decision-making across the intervention’s life (Kok et al., 2016, 2017), researchers may selectively choose steps of the intervention mapping that apply to the study context (Fernandez et al., 2019). This study adapted the process to employ only the first four steps as part of the Project SMART PA intervention design.

### Step 1: Defining the Problem

**FIGURE 1:**
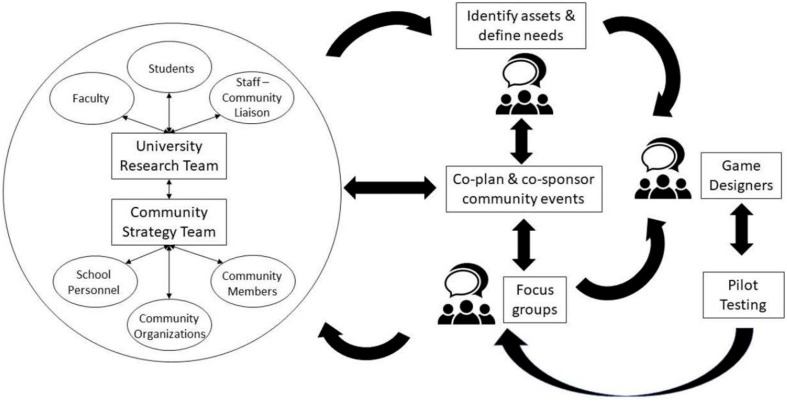
Community-based participatory research as a university-school-community partnership.

The **first step** of the IMP was to establish a planning group and conduct the needs assessment. Using a community participatory research approach, the planning group brought together university researchers from multiple disciplines (engineering, health education, and kinesiology), teachers, students, and community partners to co-design the Project SMART game (Figure 1). The advantage of using a community participatory research process is that it involves researchers and stakeholders working together to understand a problematic situation and change it for a better community (Baum et al., 2006; Kemmis et al., 2013). This process usually focuses on social change that promotes behaviors and challenges inequality. Since the approach is context-specific, it is focused on the needs of a particular group—in this case, elementary school children. Through this process, the school community members and research team share in the decision-making, instead of the researchers making all decisions and asking the school community members for input after decisions have already been made. Needs assessment data came from one student focus group interview, subjective and objective PA data, and the Fitness Education Index. All needs assessment data would diagnose what barriers are encountered in the current school settings to promote PA using the SMART Project.

#### Student Focus Group

As the first step, one student focus group interview was conducted to investigate the students’ needs related to PA motivation. The student focus group participants included two university engineering majors, two university faculty, twenty-two 4th grade students and their teacher. Based on KidsGoGreen (Farella et al., 2020), used in Italy to motivate sustainable transportation to school, the development of Project SMART was initiated as a final project for electrical and computer engineering students at The University of Texas at Austin. With the engineering majors acting as the session facilitators, they introduced their idea to create an online PA game. In being introduced to the online PA game project, the 4th grade students were asked what would engage and motivate them to participate in PA driven by traditional education. Although the engineering majors had created a semi-structured interview protocol for the in-school focus group, the 4th graders were so engaged that the event turned into an interactive brainstorming session, where the research team gathered ideas on how to promote PA through online PA games. Noted answers were used as resources for the development of the protocol. Two observers recorded notes and axial coded the responses for patterns, using team debriefing sessions.

#### Student Objective Physical Activity Data

Developing accurate and reliable tools for quantifying PA data in school-aged children continues to be a research priority. Five to seven days of PA data provide reliable estimates of usual PA behavior in children because it accounts for recurring patterns of inactivity (Trost et al., 1999). PA data are necessary for studies designed to identify the effectiveness of programs to increase PA in school-aged children (Clark et al., 2017). Five different groups totaling 167 children, 9–12 years of age, from five different elementary schools in Central Texas participated in the PA data collection. The type of schools included: (a) one charter school (tuition-free public schools), (b) three Title 1 schools where at least 50% of the students were from low-income families and demonstrated low levels of academic achievement, and (c) one academically high performing school. Four of the five schools were minority-majority, ranging from 40–93% Hispanic students. Objective PA data collection took place during six 45-min physical education lessons, using self-reported and objective data from wearable devices. PA data were collected during physical education because students were participating in the same structured activities, which would enable the physical education teacher to discuss various levels of PA and how they apply in the lesson. It also allowed data collectors to observe many students to better determine whether they were accurately reporting their PA levels. Once IRB and school permissions were granted, the participants were asked to wear an accelerometer (GT3X accelerometer; Actigraph^TM^, LLC, Pensacola, FL, United States) to collect data in five-second epochs during physical education classes, which were held two times per week. Students were familiarized with how to wear the accelerometer by both the teacher and the researchers present on all data collection days to make observations, complete fidelity logs, and assist with the PA data collection.

#### Student RFID Self-Reported Physical Activity Data

**FIGURE 2:**
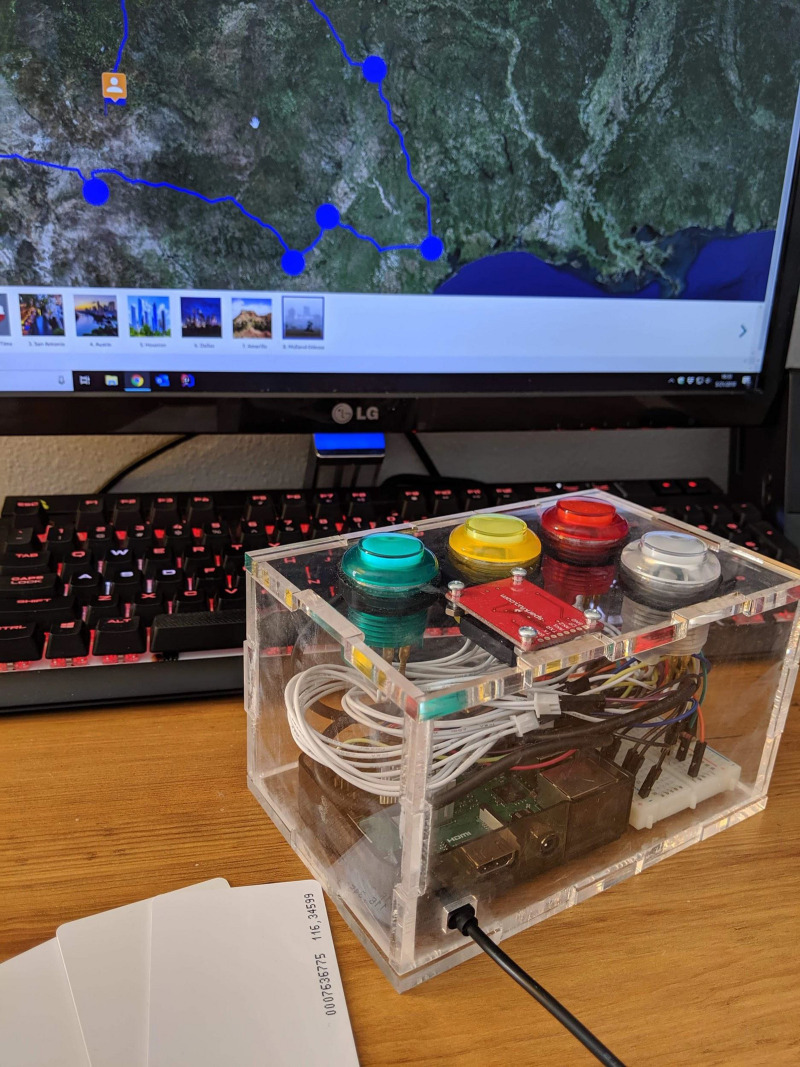
SMART box for RFID badge and self-report of physical activity. The figure is reused from Julien et al. (2021) with permission.

In the second round of PA data collection, the students were asked to report the intensity of their PA during the physical education lesson through a PA badge system. Using the Project SMART check-in box (Figure 2), the students swiped an RFID badge to check-in and then selected the green (very active), yellow (active), red (less active), or white (inactive because of non-participation or absence) button as they exited the gym to record their PA intensity for the lesson. The researchers then compared self-reported PA data with their objectively collected PA intensity on the accelerometer. Self-reported PA data collection continued for 20, 45-min physical education lessons. A direct comparison between subjective and objective measures of PA were made for three randomly selected classes that provided insight into students’ misconceptions about PA intensity, behavioral compliance, and teacher expectations.

#### Fitness Education Index

A teacher self-assessment utilized the Fitness Education Index (Chen et al., 2020). The Fitness Education Index is a 20-item self-assessment scale to determine an organization’s readiness to provide PA instruction. The assessment tool was adapted from the School Health Index [Centers for Diseases Control and Prevention (CDC), 2014] and used the indicators of *not in place*, *underdevelopment*, *partial implementation*, and *full implementation* of the factors of PA programming, nutrition programming, instructional strategies, standards alignment, professional development, and family/community engagement. In preparation for developing this intervention, researchers contacted schools in Central Texas to quantify organizational readiness and allow Project SMART to be responsive and flexible to the organizational needs. Specifically, 21 area physical education teachers completed the Fitness Education Index. The sum score on the index represented the degree of readiness. This scale is a needs assessment to determine the readiness for the PA intervention from a school level.

### Step 2: Identifying Program Outcomes and Performance Objectives

The **second step** was to identify expected outcomes of the intervention and formulate developmentally appropriate performance objectives (i.e., sub-behaviors within the desired behavior) at each level for behavior change. For example, to achieve expected outcomes, an individual would need to take many actions, including moving more in the classroom, monitoring PA level using technology, and avoiding prolonged periods of sedentary behaviors. As mentioned earlier, in the aims of this project, the desired outcomes were to increase PA levels in and out of school and the surrounding community. However, it was recognized that PA could arise in several different contexts (e.g., during classroom activity, physical education lessons, and before/after school) and have many environmental influences (e.g., personal, interpersonal, and environmental). Therefore, researchers conducted adult focus group interviews to identify each context’s expected outcomes and each level of influence aligned with CSPAP components. From these data, we created the conceptual model for Project SMART.

#### Adult Focus Groups

Using a community participatory research approach, we asked teachers, parents, and community members (other program organizers) to participate in focus groups. Also, we engaged teachers in focus groups to understand the school’s climate and its members and identify relevant people in the community and how they approach learning in the 4th and 5th grades. Specifically, four focus groups were conducted: (a) two at a WCWH community event and (b) two exclusively with teachers using online teleconferencing involving a total of 22 teachers. In addition, the focus groups involved 10 community members who elected to attend a community wellness fair co-sponsored by WCWH and the UT Austin School of Nursing. The focus of the event was on family wellness and information access. In a wellness fair-like atmosphere, families stopped at tables and were asked in small groups to answer questions about their *points of pride* and *concerns* about children’s health in this community. Note that the teacher focus groups were conducted during the COVID-19 pandemic, at the end of the school year, after school campuses had closed and teachers were asked to deliver remote learning without any preparations. Moving to remote learning likely influenced our findings and guided us in moving forward with attainable objectives.

### Step 3: Selecting Theoretical Methods and Practical Application

The **third step** was designing a program aligned with theoretical methods and practical strategies. A theoretical approach is a general process intended to change behavioral determinants (e.g., self-efficacy). Practical application refers to the way such a theoretical method is put into practice in the actual classroom. Here, researchers chose the Self-Determination Theory (SDT) constructs to connect Project SMART game modules to learning activities intended to increase intrinsic motivation for participation in PA. Many interventions use the SDT to improve PA levels by motivating school-aged children (Gillison et al., 2019; Vasconcellos et al., 2020). This motivational theory has been widely used in recent years to develop interventions aiming to improve student motivation in multidimensional contexts such as physical education lessons (Cheon and Reeve, 2015), classrooms (Sergis et al., 2018) and extracurricular programs (González-Cutre et al., 2018). However, few studies undertook an intervention with students from minority groups, and the program was disconnected from their needs and interests (Fahlman et al., 2015). Project SMART engaged children, teachers, parents, and staff from socioeconomically disadvantaged neighborhoods as co-researchers in the community participatory research. The SDT (Ryan and Deci, 2000) provides a theoretical framework that plays an essential role in motivating students to have a physically active lifestyle. Our study applies this framework in the elementary school settings to help readers utilize the theory in real-world scenarios with students. For example, relatedness is addressed by encouraging students to ask questions, engaging in authentic co-design and gameplay, and continuously providing student with peer and role model support. Challenging yet achievable goals have been established, and students are encouraged to self-monitor to increase competence regarding PA and game participation (Teixeira et al., 2020). Also, the co-researchers examined the self-reported PA data across 20 physical education lessons. A time series analysis was used to understand the nature of the observations and to explain the PA monitoring. Findings from the ARIMA modeling analysis (Dixon, 1992) of the repeated measure PA data collection are presented.

### Step 4: Program Production and Implementation

Refining the program structure and identifying outcomes for program implementation were part of the fourth step. This step includes defining and describing the learning activities of the intervention and program protocol. Together the inputs, activities, and short-, intermediate- and long-term outcomes for Project SMART were identified and conceptualized in the logic model.

## Results

### Step 1: Defining the Problem

To establish a participatory planning group, researchers engaged the community, springboarding off the WCWH initiative and establishing a research team of community members, teachers, and university researchers to serve as co-investigators. This section presents an overview of the needs assessment data through four different resources.

The **student focus** group found the 4th grade students highly engaged and motivated by the potential of contributing to the design of an interactive, educational game. The students reported that they wanted gamification elements of competition, levels, leaderboards, and avatars, while the teacher suggested that the game be cooperative and driven by the aggregate class PA so that no student could be singled out as a non-contributor or identified as falling behind the others. Once the idea of cooperation was introduced to the students, they were agreeable to the recommendation brought forward by the teachers. As a result, the research team decided to have a cooperative but individually propelled PA game. The teacher suggested that the game had to be directly aligned with the grade-level educational learning outcomes in Science, Technology, Engineering, and Math (STEM) and those outcomes for social-emotional learning (SEL), cultural studies, and physical education. Several teachers and school administrators would later confirm, the intervention would be a non-starter if the educational standards were not included because they were under pressure to use all academic time to focus on achievement.

**Objective PA** data revealed that none of the 20 observed physical education lessons achieved the recommended standard of 50% of the class time or at least 23 min in MVPA [Office of Disease Prevention and Health Promotion (ODPHP), 2019]. Only three specific lessons resulted in a class average of approximately 40% or higher of time in MVPA. Lesson content significantly influenced the amount of MVPA during a class session (Health-related fitness lesson M MVPA = 12.56 min; SD = 8.00; Low organizational games lesson M MVPA = 12.32 min; SD = 1.99; Motor skills lesson M MVPA = 17.67 min, SD = 6.08; *F*(2, 139) = 10.09, *p* < 0.001). Surprisingly, the health-related fitness lessons that employed fitness stations and a circuit training format did not produce the highest MVPA. Instead, the lessons focused on motor skills, where each student had their own equipment and space to repeatedly practice resulted in the highest MVPA. Although contrary to previous research (Nader et al., 2018), the type of school or teacher was not significantly related to the amount of MVPA. This is likely because we only worked with physical education teachers who were already known to be conscientious professionals. Therefore, differences across the schools were not in the teacher’s approach but instead potentially related to contextual variables (e.g., high and low academically performing students).

**FIGURE 3:**
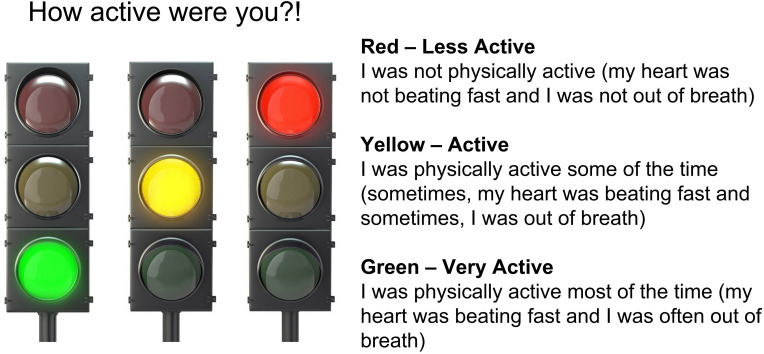
Visual of how to self-report physical activity intensity.

**Self-reported PA** intensity data collected using an RFID badge can be found in Table 1. Initially, the 4th graders were only accurate approximately one-third of the time and tended to overestimate the intensity and total volume of PA (58%) but did improve once given feedback from the accelerometers. Students in the 5th grade underestimated (46%) their PA intensity and volume and instead reported if they followed the rules and had listened to the teacher. Students’ self-reported PA was related to behavioral compliance and listening to the teacher more than participating at moderate to vigorous intensity. These developmentally appropriate responses are rarely considered during the design of a PA intervention. PA intensity is related to the magnitude of health benefits. As such, it should be introduced as an educational outcome of the intervention (e.g., jumping rope made me sweat, but walking on the balance beam did not). A Chi-Squared analysis *X*^2^ (2, 90) = 12.83, *p* = 0.002 revealed that 5th graders were significantly more accurate than 4th graders, as would be developmentally expected. There was no significant difference between time one and time three concerning the accuracy of reporting of PA intensity for all students. However, a paired sample *t*-test of just 5th graders revealed a significant improvement in accuracy for time one and time three, *p* < 0.05, suggesting a potential learning effect. For example, between time one and time two assessments of PA reporting accuracy, we added a traffic light poster that included descriptions defining each color (Figure 3). Further, the teacher stood nearby during the check-out, so the students had independent time to carefully consider their selection. Finally, as a co-investigator, the teacher suggested offering an SEL lesson focused on honesty in PA settings.

**TABLE 1:** Objectively measured physical activity versus student reported physical activity.

Grade	Time 1—Low Organizational Games (*n* = 90)	Time 2—Motor Skills, Basketball Dribbling (*n* = 86)	Time 3—Health-Related Fitness Circuit Training (*n* = 80)
Grade 4 (*n* = 45)	29% Accurate 58% Overestimated 13% Underestimated	39% Accurate 46% Overestimated 15% Underestimated	40% Accurate 45% Overestimated 15% Underestimated
Grade 5 (*n* = 45)	25% Accurate 29% Overestimated 46% Underestimated	50% Accurate 15% Overestimated 37% Underestimated	63% Accurate 12% Overestimated 25% Underestimated

The **Fitness Education Index** analysis was based on the sum score from the 21 teachers representing the degree of readiness (*M* = 40.15; SD = 9.58). The index revealed that eight schools were already engaged in the delivery of CSPAP components. However, with 13 schools still a work in progress on implementing PA opportunities across the school day (e.g., before/after school, recess, PA in the classroom, daily physical education), the research team needed to plan on providing more support for the schools for successful implementation of a PA intervention. The resulting decision was to provide teacher professional development, familiarization to Project SMART, and have the students run the game while the teachers concentrated on delivering content. Further, to engage multiple components and levels simultaneously, the researchers mapped the CSPAP component, performance objectives, SDT constructs to help the school personnel identify who was best positioned to integrate that portion of the intervention (Table 2).

**TABLE 2:** Example of CSPAP component, performance objectives, and SDT constructs.

Program outcomes	Performance objectives students/Teachers	Autonomy	Relatedness	Competence
Increase student physical activity during physical education lessons	• Actively engage in quality physical education lessons • Monitor physical activity intensity	• Develop lessons that facilitate student choice • Facilitate student decision-making in physical activity settings	• Set individual and class goals • Cooperate with classmates to achieve a common goal	• Monitor physical activity participation • Achieve grade-level physical education learning outcomes
Increase student physical activity participation during the day	• Choose to engage in more physical active during recess and classroom breaks	• Play with interest • Make healthy choices	• Build systems of support • Enjoy movement • Perceived caring	• Confidently move • Practice healthy-decision making
Increase student’s physical activity before/after school	• Participate in more physical activity outside of school	• Choose to participate in physical activity during free time	• Communicate their wish to participate in physical activity	• Confidently demonstrate motor skills • Perceived ability
Increase teacher physical activity promotion	• Integrate physical activity into the daily plan	• Empower students to make healthy choices	• Student-centered approach to learning • Use physical activity data to understand STEM content	• Achieve STEM learning outcomes • Mastery of academic skills like problem solving and collaboration

### Step 2: Identify Program Outcomes and Performance Objectives

The current intervention’s primary outcome was to increase the student’s PA level during the school day. Here, the researchers formulated the expected outcomes within CSPAP components and identified the performance objectives to develop a logic model. As the first process, a focus group interview was conducted with the community and school stakeholders.

The **adult focus groups** resulted in the formulation of three themes. First, the researchers learned that the community, made up of four neighborhoods, was unincorporated and relied heavily on schools for governance and guidance. “We trust our teachers and the nurses at the clinic,” stated one parent (which was later corroborated across multiple data sources). This is a common problem in suburban and rural communities with no main street or large chain stores as its centerpiece (e.g., Walmart). The second theme that emerged before, during, and beyond the pandemic was the lack of reliable high-speed Internet access. The school district reported through attendance records and weekly teacher reports that in Spring 2020, one-third of total district enrollment did not have access to the Internet, and therefore thousands of school-aged children were not able to participate in remote learning activities. Of particular concern were families with children in grades PreK-2, who did not initially receive a school district device (i.e., Chromebook).

Further, as reported in a five-question survey of 76 heads of households in this community, families only had one device, typically a cell phone, which inhibited them from accessing health information and resources, like time and locations for food bank distributions. Since reporting these findings, mobile hotspots and Chromebooks have been deployed to all families with at least one child registered with the school district. The last theme was a concern about the lack of PA and obesity among children. In general, there were misconceptions about how much PA children get during a school day when on campus. When asked how much PA their children were participating in, the consensus was that the schools took care of that on the weekdays and that the family was responsible for engagement on the weekends. As a result, families did not have access to PA opportunities or information about being active as a family.

Based on the themes, the research team decided that the expected outcome would be to “increase PA” within the CSPAP framework during school hours. It was determined that focusing on PA within the home and school environment at the onset of the intervention would be overwhelming. It is currently recommended that children participate in 60 min or more of MVPA daily (Pate and O’Neill, 2008); however, it was recognized that it was an unrealistic goal for physical education classes alone. Therefore, it was decided that each CSPAP component would play an independent but valuable role in the intervention. For example, a school should provide at least 20 min of recess per day, in addition to a physical education class that allows participating in free-time PA and practice skills learned in physical education lessons. Also, recess is typically delivered in an outdoor, unstructured environment, which may favor engagement of at least 50% of recess time in MVPA (Stratton and Mullan, 2005; Tran et al., 2013). The goals were relative to the individual and the class—overall, PA should significantly increase from baseline levels.

To meet our objectives, the students need to act on intrinsic motivation to help them consider how and when to move more and sit less (Gardner and Lally, 2013). Therefore, the expected outcomes focused on increasing the rate of PA participation required meeting the three psychological needs of students’ motivation (i.e., autonomy, relatedness, and competence) within CSPAP components. Next, performance objectives for each of the program outcomes should be specified. This step requires what needs to happen to influence the expected outcomes (Table 3). Finally, the team aligned the performance objectives in the theoretical determinants of motivation with each CSPAP component.

**TABLE 3:** Motivation and behavior change techniques.

Research and game elements	Self-determination theory		

Researcher inputs	Autonomy	Relatedness	Competence
Motivation and behavior change techniques (Teixeira et al., 2020) Points of intervention: Classroom, physical education, recess, and home	• Encourage students to explore and share perspectives • Provide choice • Encourage students to self-initiate PA and gameplay	• Encourage students to ask questions • Invite student feedback • Provide continuous student support	• Establish challenging yet achievable goals • Provide focused learning activities • Develop equitable educational content

**Student outcomes—game play**	**Autonomy**	**Relatedness**	**Competence**

Physical Activity Participation Points of Intervention: Classroom, physical education, recess, and home	• Choose type and intensity of physical activity • Play of interest • Develop enjoyment for physical activity	• Peer encouragement • Relationship building • Student-centered approach • Empowerment in physical activity settings	• Success in learning outcomes (e.g., skill and performance) • Confidence in movement • Practice healthy decision making • Confidence in motor skills
Monitoring Physical Activity Participation	• Choose to log activity • Enjoyment in-game engagement	• Peer encouragement • Similar goals and experiences with peers	• Success in-game navigation • Confidence in-game engagement • Ability to self-pace
Cooperative Play	• Choose to play • Choose to support others in a shared goal	• Teamwork • Peer encouragement • Shared experience	• Contributing to class accomplishments in gameplay • Strength-based contribution

**Student Outcomes—Modules^a^**	**Autonomy learning activity**	**Relatedness learning activity**	**Competence learning activity**

Visiting Atlanta, GA K12CS Grades 3–5 standards • Computing systems • Data storage • Data collection • Data analysis and visualization	The students learn basic coding while allowing for individual creativity and choice. • Choose what data to collect (temperature or time) • Store collected data • Visual display the data	The students describe an idea as a different representation. • Several movies were filmed in Atlanta. Use a character in a movie to build identity as a scientist	The students refine map reading skills. • Using a map of public transportation in Atlanta, students follow instructions to a destination • Students map Atlanta railroad transportation in 1840s versus today

*^a^There are over 30 learning activities embedded in the game. We provided examples to demonstrate how theory was mapped onto the computer science and STEM learning outcomes for students and integrated into the school curriculum and daily activities.*

### Step 3: Select Theoretical Methods and Practical Application

A review of the literature confirmed that the SDT was likely the most appropriate for Project SMART. This intervention requires motivation, and because the SDT is focused on that construct and the principles of three innate psychological needs for autonomy, competence and relatedness (Ryan and Deci, 2000), it was selected. Autonomy refers to behavior that is self-endorsed that people agree with and find congruent within themselves. Students have autonomous motivation when they are interested and enjoy the activity. The autonomous experience is a full set of volition, willingness and choice about what students are doing at the moment. Relatedness is feeling cared for and connected to others. It has to do with a sense of belonging and a feeling that one’s matters to others. Relatedness is enhanced not just by people treating a person warmly, but also by one’s giving to them, and one can matter in their lives that is part of what helps us feel connected. So, it is not one-way, but it has to do with bidirectional connections. Competence is essential to physical wellness to feel useful in people’s environment to have some sense of mastery of essential things to people. In contrast, when these psychological needs are not fulfilled, people regulate their behavior based on controlled reasons (Karagiannidis et al., 2015).

Self-Determination Theory also postulates that human behavior in any context can be intrinsically motivated, extrinsically motivated, or amotivated. Intrinsic motivation is evident when individuals freely engage in activities they find interesting and enjoyable and offer the opportunity for learning or task accomplishment (Pelletier et al., 1995). In contrast, extrinsic motivation is apparent when individuals perform an activity because they value their associated outcomes, such as public praise and extrinsic rewards, more than the activity itself. A lack of motivation refers to the shortage of intrinsic and extrinsic motivation and represents a complete lack of self-determination and volition concerning the target behavior (Deci and Ryan, 1985; Ryan and Deci, 2000). For example, an unmotivated student may feel that physical education does not serve any purpose and may exhibit boredom, low attendance, or passive participation in the lessons. Therefore school-based PA interventions should consider how to stimulate intrinsic motivation rather than unmotivated students surrounding the school. Supporting SDT with three psychological needs is strongly correlated with autonomous student motivation in PA environments (Vasconcellos et al., 2020). Project SMART applies motivation and behavior change techniques (Teixeira et al., 2020), encouraging students to explore and share their perspectives, provide meaningful rationales to be physically active, and urge students to self-initiate PA and gameplay to increase intrinsic motivation (Table 3).

**FIGURE 4:**
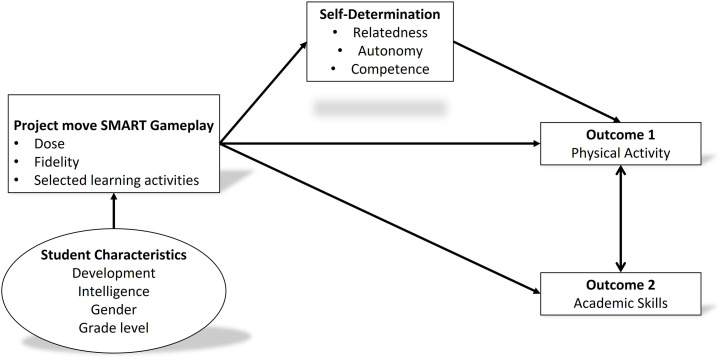
Project move SMART conceptual model.

Growing evidence supports the implications of SDT for health behavior change. For example, SDT-based research has shown that more self-determined regulations can predict PA engagement adherence (González-Cutre et al., 2018). In addition, in educational settings, gamification and gaming elements can help students change behavior by supporting the basic psychological needs for autonomy, competence and relatedness (Kim and Castelli, 2021). In the previous step, the performance objectives were created seeking answers to such questions as, “What PA should I choose to do on my own time?” and “What activity do I like and feel comfortable playing?” These questions target the SDT determinants of autonomy, competence, and relatedness. After identifying the performance objectives and determinants of behavior change, the research team developed the logic model–what students have to learn to accomplish those objectives through change objectives. To guide the research team through this step of the process, members created a conceptual highlighting of the causal pathway between gameplay and increased PA and increased academic skills mediated by self-determination (Figure 4).

**FIGURE 5:**
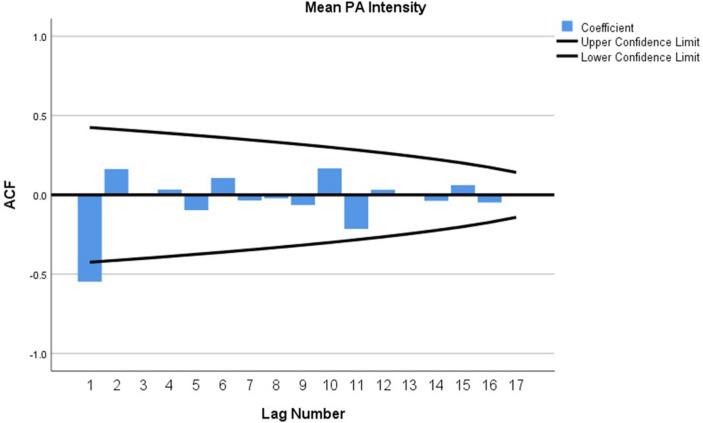
Confidence interval limits of self-reported physical activity data.

The research team tested how Project SMART gameplay can be applied in an SDT-based framework as an example of this conceptual model’s practical application. The participants were asked to **self-report PA intensity** when they participated in a physical education class. The students could report data from their class and were encouraged to report PA beyond that experience (e.g., recess, walking home from school, PA in the home environment) as part of the gameplay. Because this request produced a set of 20 repeated observations or the same variable over 10-weeks, SPSS v.26 was used to conduct a time series of measures to forecast stability of responses and potential regression toward the mean through Autoregressive Integrated Moving Average (ARIMA modeling: Dixon, 1992; Cromwell et al., 1994). No single reported score was more than two standard deviations away from the mean, suggesting no outlying scores were included in the final analysis. In Figure 5, when the value is closer to one (*d* = 1), it means the series is not stationary, which is reflected in the data across the first 6 weeks, but from that point forward, you see a *d* = 0, which means that the series is stationary and that children have increased and are currently maintaining their PA intensity to report participation as “active” or “very active” on these days. Autocorrelations were calculated using the time series observations and observations in the previous step, called lags. The analysis revealed no significant serial correlations in 15 of the 20 observations, suggesting that self-reported PA is a stable, reliable construct predicting intensity. Further, these data indicate that after PA increased from baseline, they were maintained over 10 weeks following, regardless of physical education content or the number of opportunities at school.

Given these results and the application of SDT, a team of teachers, university students, and faculty began building the learning modules. Aggregate class PA unlocked learning activities associated with SDT constructs and STEM and SEL grade-level outcomes. The team attempted to increase perceived competence in STEM learning that includes moving. It was hypothesized that increased perceived competence would be a transferable academic achievement (Ntoumanis, 2001).

Each psychological need is relevant from the perspective of SDT and was integrated into each learning module. Specifically, autonomy was targeted through activities that students found interesting and enjoyable. The modules gave students a choice in what actions to take and when—virtually traveling across the country gave students ownership of their learning. The student-centered and strength-based approach brought a new level of relatedness to healthy decision-making (e.g., Should we stop and complete the module that will provide us with water for our journey?). Many of the learning activities are scenarios and web-based applications to increase relatedness. Competence is enhanced through progress bars reflecting achievement of goals, completion of modules and rewarding of badges. The opportunities to practice healthy decision-making within a supportive environment have merit. Unconventionally, on their journey, 5th graders will encounter an outbreak of malaria in Jamestown, Virginia, while 4th graders will compare census data from 1850 and 2010 to explain the environmental impacts of a growing population. The contextual and pedagogical approaches of addressing all STEM disciplines simultaneously include using class PA and location data, assessing for learning, alignment with learning state and national standards, and integrating historical, cultural, and geographic facts, ***making this intervention both novel and timely.***

### Step 4: Program Production and Implementation

During this step, Project SMART intervention pilot testing was performed in selected elementary schools. Educational game elements structure the Project SMART to optimize the motivation to increase the PA level for children. Previous research has shown that games have a notable motivational factor; they utilize several mechanisms to encourage students to engage with them, often without any reward, just for the joy of playing (Cronk, 2012; Beça et al., 2020). This is also aligned with intrinsic motivation. Participation in the game teaches and reinforces knowledge and develops essential skills such as problem-solving, collaboration, and communication (Hamari et al., 2014; Dicheva et al., 2015). Recent studies have adopted game elements such as exergaming and virtual PA using technologies to promote children’s PA. The emerging game element using technologies is called *gamification.* Gamification has been used to support user engagement and enhance positive service use patterns, such as increasing user activity, social interaction, or quality and productivity of action (Hamari, 2013). The process of gamification is an innovative pedagogical approach employing game design elements in non-game contexts is a relatively new and rapidly growing field (Hamari et al., 2014; Chou, 2019). This approach has been integrated into an educational context to address social behavior and student motivation problems. Therefore, in Project SMART the researchers combined both gamification and SDT framework to build a motivating educational module system to increase the PA level.

## Discussion and Implications

The Project SMART intervention is one of the first attempts to develop the program in ethnic minority groups that integrate SDT components using IMP through community-based participatory research. Focus group data indicated that children faced several barriers in school, in particular, lack of interest regarding PA made it hard for students to motivate engaging in PA. Project SMART uses techniques to increase student motivation to participate in PA while also encouraging their classmates to assist in progressing them along their journey. The researchers utilize positive and non-judgmental language to discuss PA and educational goals (Teixeira et al., 2020) while also asking them to provide feedback to help shape the game. Further, obstacles reported by students and teachers have been addressed (e.g., need for PA materials at home and alignment with educational goals), and researchers aim to increase confidence in promoting and playing the game.

Some implications can be found from the objective- and self-reported PA data. First, MVPA was significantly influenced by lesson content, not the type of school or teacher. When the team examined the lesson content in detail, students were more active when performing their motor skills with their own equipment and space with enough practice time. These results implied that students could improve their PA level when they have higher components of SDT in students’ perception. Second, self-reported PA was gradually improved when students received feedback from objective data and teacher instruction. Correction of student’s awareness regarding PA can improve PA levels that are important for the educational goal. Therefore, teachers can use various resources to enhance traditional teaching methods and keep students more engaged in using technology, improving students’ learning. Third, each grade has different educational goals that require different developmental trajectories. The pilot data showed that the self-reported PA between the 4th and 5th grades was significantly different. Therefore, when teachers plan lessons, it is recommended to align with national and state standards by grade level.

Previous research examined the high levels of obesity, especially among ethnic minority and low-income students who have less access to well-resourced PA opportunities and less family support for participating in PA (Molnar et al., 2004; Wilson et al., 2011). Similarly, as shown in the adult’s interview, not every student had access to web-based learning. Students who have less access to academic opportunities would have a greater chance of missing academic improvement. Schools, especially in minority communities, should consider providing a “whole-person approach” and overcoming those “barriers” that will eventually impact children’s health and academic achievement. The team believes a theory-based practical intervention effectively increases MVPA in low-income and minority students during Project SMART, but further research is needed to address home barriers to children’s MVPA. There are limitations to the study that should be noted. As a process of the evaluation study, the PA estimates and interview data are contextually and culturally grounded and are insufficient to generalize to United States schools. For example, the RFID badge system has three options for PA intensity, and this self-reported PA might not determine the dimensions of students’ PA level. However, our IMP methods allowed us to utilize the maximum amount of information to evaluate school circumstances in minority and low-income communities. Further research is needed to expand the current project for capturing missing values to improve the PA level in school.

## Summary

This paper describes the Project SMART intervention’s systematic development using modified IMP and community-based participatory research methodology. Four steps of intervention development required from focus group interviews, PA data, and Fitness Index data to program design. The process of program development is based on the theoretical framework that applied SDT. The aggregation of pilot data-driven builds as the logic of Project SMART intervention. Utilized as an educational game, elements such as virtual journey from students’ objective PA motivate students to increase PA while achieving STEM and SEL learning standards. Since developing interventions systematically increases the likelihood of effectiveness (Brug et al., 2005), this development process represents an essential strength. The study also aims at providing essential insights into children’s intrinsic motivation that can be elicited by the SMART game. To promote PA in school effectively, students need to possess intrinsic motivation that stakeholders, including teachers, staff, and parents, should plan to improve autonomy, relatedness and competence for PA participation.

Although time-consuming, the community-based participatory approach has already yielded numerous benefits and unintended outcomes, such as new collaborations and sharing resources, tighter research design “fitted” to the context and appropriate goals. Moreover, a transdisciplinary research team utilized knowledge from multiple disciplines and engaged citizens, who typically are not involved in research, in science as co-planners. Thus, the idea of citizen science has potential impactful and far-reaching effects, but most are beyond the scope of this study.

The Project SMART intervention is only now being fully implemented, it is expected that children who participate in Project SMART will demonstrate greater PA-level improvements than children following the standard school curriculum. It is also expected that children in Project SMART will show greater academic engagement and academic performance than children in the school’s regular curriculum. Furthermore, children in Project SMART will demonstrate greater gains across self-determination elements (autonomy, competence, and relatedness) to the PA among children in the control group. Although a long process, the intervention using IMP allowed us to develop a theory-based practical intervention that can be delivered in elementary schools.

## Data Availability Statement

The raw data supporting the conclusions of this article will be made available by the authors, without undue reservation.

## Ethics Statement

The studies involving human participants were reviewed and approved by The University of Texas at Austin. Written informed consent from the participants’ legal guardian/next of kin was not required to participate in this study in accordance with the national legislation and the institutional requirements.

## Author Contributions

YJ: conceptualization, planning, protocol development, data analysis, and writing of the manuscript. SB: planning, data collection, data analysis, and writing of the manuscript. CJ: conceptualization, planning, supporting proofreading, and supporting data analysis. DB: protocol development, supporting proofreading, and data analysis. DC: conceptualization, planning, data analysis, and writing of the manuscript. All authors contributed to the article and approved the submitted version.

## Conflict of Interest

The authors declare that the research was conducted in the absence of any commercial or financial relationships that could be construed as a potential conflict of interest.

## Publisher’s Note

All claims expressed in this article are solely those of the authors and do not necessarily represent those of their affiliated organizations, or those of the publisher, the editors and the reviewers. Any product that may be evaluated in this article, or claim that may be made by its manufacturer, is not guaranteed or endorsed by the publisher.

## Footnotes

1
https://bridgingbarriers.utexas.edu/whole-communities-whole-health/

2
https://tea.texas.gov/academics/curriculum-standards/teks/texas-essential-knowledge-and-skills

## References

[B1] Barbosa Filho V. C. Minatto G. Mota J. Silva K. S. de Campos W. Lopes A. S. (2016). Promoting physical activity for children and adolescents in low- and middle-income countries: an umbrella systematic review: a review on promoting physical activity in LMIC. *Preven. Med.* 88 115–126. 10.1016/j.ypmed.2016.03.025 27068650

[B2] Bartholomew-Eldredge L. K. Markham C. M. Ruiter R. A. Fernández M. E. Kok G. Parcel G. S. (2016). *Planning Health Promotion Programs: An Intervention Mapping Approach*, 4th Edn. San Francisco, CA: Jossey Bass.

[B3] Basch C. E. (2011). Physical activity and the achievement gap among urban minority youth. *J. School Health* 81 626–634. 10.1111/j.1746-1561.2011.00637.x 21923875

[B4] Bates L. C. Zieff G. Stanford K. Moore J. B. Kerr Z. Y. Hanson E. D. (2020). COVID-19 impact on behaviors across the 24-Hour day in children and adolescents: *physical activity, sedentary behavior, and sleep*. *Children* 7:138. 10.3390/children7090138 32947805 PMC7552759

[B5] Baum F. MacDougall C. Smith D. (2006). Participatory action research. *J. Epidemiol. Community Health* 60, 854–857. 10.1136/jech.2004.028662 16973531 PMC2566051

[B6] Beça P. Aresta M. Ortet C. Santos R. Veloso A. I. Ribeiro S. (2020). “Promoting student engagement in the design of digital games: the creation of games using a Toolkit to Game Design,” in *2020 IEEE 20th International Conference on Advanced Learning Technologies (ICALT)*, (IEEE), 98–102.

[B7] Biddle S. J. H. Ciaccioni S. Thomas G. Vergeer I. (2019). Physical activity and mental health in children and adolescents: an updated review of reviews and an analysis of causality. *Psychol. Sport Exercise* 42 146–155. 10.1016/j.psychsport.2018.08.011

[B8] Breny J. M. (2020). Continuing the journey toward health equity: becoming antiracist in health promotion research and practice. *Health Educ. Behav.* 47 665–670. 10.1177/1090198120954393 32896177

[B9] Brug J. Oenema A. Ferreira I. (2005). Theory, evidence and Intervention Mapping to improve behavior nutrition and physical activity interventions. *Int. J. Behav. Nutr. Phys. Activity* 2:2. 10.1186/1479-5868-2-2 15807898 PMC1087867

[B10] Brusseau T. A. Hannon J. Burns R. (2016). The effect of a comprehensive school physical activity program on physical activity and health-related fitness in children from low-income families. *J. Phys. Activity Health* 13 888–894. 10.1123/jpah.2016-0028 27144329

[B11] Burns R. D. Brusseau T. A. Hannon J. C. (2015). Effect of a comprehensive school physical activity program on school day step counts in children. *J. Phys. Activity Health* 12 1536–1542. 10.1123/jpah.2014-0578 25741980

[B12] Castelli D. M. Centeio E. E. Nicksic H. M. (2013). Preparing educators to promote and provide physical activity in schools. *Am. J. Lifestyle Med.* 7 324–332. 10.1177/1559827613490488

[B13] Centers for Disease Control and Prevention (CDC) (2012). *Step 3: Focus the Evaluation Design.* Available online at: https://www.cdc.gov/eval/guide/step3/index.htm (accessed December 17, 2020).

[B14] Centers for Diseases Control and Prevention (CDC) (2014). *School Health Index (SHI): Self-Assessment and Planning Guide.* Atlanta, GA: Centers for Disease Control.

[B15] Chang C. D. (2019). Social determinants of health and health disparities among immigrants and their children. *Curr. Prob. Pediatric Adolescent Health Care* 49 23–30. 10.1016/j.cppeds.2018.11.009 30595524

[B16] Chen Y. T. Barcelona J. M. Cance J. D. Calvert H. G. Barnes S. P. Wargo J. (2020). Development of the fitness education index: a scale of organizational level capacity. *Res. Q. Exercise Sport* 91 172–178. 10.1080/02701367.2019.1654066 31617835 PMC7183731

[B17] Cheon S. H. Reeve J. (2015). A classroom-based intervention to help teachers decrease students’ amotivation. *Contemporary Educ. Psychol.* 40 99–111. 10.1016/j.cedpsych.2014.06.004

[B18] Child and Adolescent Health Measurement Initiative (CAHMI) (2019). *2016-2017 National Survey of Children’s Health (NSCH) data query.* Data Resource Center for Child and Adolescent Health supported by Cooperative Agreement U59MC27866 from the US Department of Health and Human Services, Health Resources and Services Administration’s Maternal and Child Health Bureau (HRSA MCHB) Web site. Available online at: www.childhealthdata.org (accessed November 30, 2020).

[B19] Chou Y. (2019). *Actionable Gamification: Beyond Points, Badges, and Leaderboards.* Birmingham: Packt Publishing Ltd.

[B20] Clark C. C. T. Barnes C. M. Stratton G. McNarry M. A. Mackintosh K. A. Summers H. D. (2017). A review of emerging analytical techniques for objective physical activity measurement in humans. *Sports Med.* 47, 439–447. 10.1007/s40279-016-0585-y 27402456

[B21] CLIMB (2019). *KidsGoGreen.* Available online at: https://www.smartcommunitylab.it/climb-en/ (accessed November 30, 2020).

[B22] Cohen S. A. Cook S. K. Kelley L. Foutz J. D. Sando T. A. (2017). A closer look at rural-urban health disparities: associations between obesity and rurality vary by geospatial and sociodemographic factors. *J. Rural Health* 33 167–179. 10.1111/jrh.12207 27557442

[B23] Cromwell J. B. Hannan M. J. Labys W. C. Terraza M. (1994). *Multivariate Tests for Time Series Models.* Thousand Oaks, CA: Sage Publications.

[B24] Cronk M. (2012). *Using Gamification to Increase Student Engagement and Participation in Class Discussion.* 311–315. Available online at: https://www.learntechlib.org/primary/p/40762/ (accessed April 05, 2021).

[B25] Deci E. L. Ryan R. M. (1985). The general causality orientations scale: self-determination in personality. *J. Res. Person.* 19 109–134. 10.1016/0092-6566(85)90023-6

[B26] Dicheva D. Dichev C. Agre G. Angelova G. (2015). Gamification in education: a systematic mapping study. *J. Educ. Technol. Soc.* 18 75–88.

[B27] Dixon W. J. (1992). *BMDP Statistical Software Manual: To Accompany BMDP Release 7*, Vol. 1. California, CA: University of California Press.

[B28] Dobbins M. Husson H. DeCorby K. LaRocca R. L. (2013). School-based physical activity programs for promoting physical activity and fitness in children and adolescents aged 6 to 18. *Cochrane Database Syst. Rev.* 2013:CD007651. 10.1002/14651858.CD007651.pub2 23450577 PMC7197501

[B29] Eime R. M. Young J. A. Harvey J. T. Charity M. J. Payne W. R. (2013). A systematic review of the psychological and social benefits of participation in sport for children and adolescents: informing development of a conceptual model of health through sport. *Int. J. Behav. Nutr. Phys. Activity* 10:98. 10.1186/1479-5868-10-98 23945179 PMC3751802

[B30] Fahlman M. Hall H. L. Gutuskey L. (2015). Minority youth, physical activity, and fitness levels: targeted interventions needed. *Am. J. Health Educ.* 46 338–346. 10.1080/19325037.2015.1077758

[B31] Farella E. Ferron M. Giovanelli D. Leonardi C. Marconi A. Massa P. (2020). CLIMB: a pervasive gameful platform promoting child independent mobility. *IEEE Pervasive Comput.* 19 32–42. 10.1109/MPRV.2019.2939730

[B32] Fernandez M. E. Ruiter R. A. C. Markham C. M. Kok G. (2019). Intervention mapping: theory- and evidence-based health promotion program planning: perspective and examples. *Front. Public Health* 7:209. 10.3389/fpubh.2019.00209 31475126 PMC6702459

[B33] Gardner B. Lally P. (2013). Does intrinsic motivation strengthen physical activity habit? Modeling relationships between self-determination, past behaviour, and habit strength. *J. Behav. Med.* 36 488–497. 10.1007/s10865-012-9442-0 22760451

[B34] Gerosa M. Marconi A. Pistore M. Traverso P. (2015). “An open platform for children’s independent mobility,” in *Smart Cities, Green Technologies, and Intelligent Transport Systems*, eds Helfert M. Krempels K.-H. Klein C. Donellan B. Guiskhin O. (Switzerland: Springer International Publishing), 50–71. 10.1007/978-3-319-27753-0_4

[B35] Gillison F. B. Rouse P. Standage M. Sebire S. J. Ryan R. M. (2019). A meta-analysis of techniques to promote motivation for health behaviour change from a self-determination theory perspective. *Health Psychol. Rev.* 13 110–130. 10.1080/17437199.2018.1534071 30295176

[B36] Golden S. D. Earp J. A. L. (2012). Social ecological approaches to individuals and their contexts: twenty years of health education & behavior health promotion interventions. *Health Educ. Behav.* 39 364–372. 10.1177/1090198111418634 22267868

[B37] González-Cutre D. Sierra A. C. Beltrán-Carrillo V. J. Peláez-Pérez M. Cervelló E. (2018). A school-based motivational intervention to promote physical activity from a self-determination theory perspective. *J. Educ. Res.* 111 320–330. 10.1080/00220671.2016.1255871

[B38] Gordon-Larsen P. Nelson M. C. Page P. Popkin B. M. (2006). Inequality in the built environment underlies key health disparities in physical activity and obesity. *Pediatrics* 117 417–424. 10.1542/peds.2005-0058 16452361

[B39] Guthold R. Stevens G. A. Riley L. M. Bull F. C. (2020). Global trends in insufficient physical activity among adolescents: a pooled analysis of 298 population-based surveys with 1⋅6 million participants. *Lancet Child Adolescent Health* 4 23–35. 10.1016/S2352-4642(19)30323-2 31761562 PMC6919336

[B40] Hamari J. (2013). Transforming homo economicus into homo ludens: a field experiment on gamification in a utilitarian peer-to-peer trading service. *Electronic Commerce Res. Appl.* 12 236–245. 10.1016/j.elerap.2013.01.004

[B41] Hamari J. Koivisto J. Sarsa H. (2014). “Does gamification work? – A literature review of empirical studies on gamification,” in *Proceeding of the 2014 47th Hawaii International Conference on System Sciences*, (Hawaii, HI), 3025–3034.

[B42] Herlitz L. MacIntyre H. Osborn T. Bonell C. (2020). The sustainability of public health interventions in schools: a systematic review. *Implement. Sci.* 15:4. 10.1186/s13012-019-0961-8 31906983 PMC6945701

[B43] Hogan L. Bengoechea E. G. Salsberg J. Jacobs J. King M. Macaulay A. C. (2014). Using a participatory approach to the development of a school-based physical activity policy in an indigenous community. *J. School Health* 84 786–792. 10.1111/josh.12214 25388595

[B44] Jones M. Defever E. Letsinger A. Steele J. Mackintosh K. A. (2020). A mixed-studies systematic review and meta-analysis of school-based interventions to promote physical activity and/or reduce sedentary time in children. *J. Sport Health Sci.* 9 3–17. 10.1016/j.jshs.2019.06.009 31921476 PMC6943767

[B45] Julien C. Castelli D. Bray D. Lee S. Burson S. Jung Y. (2021). Project SMART: a cooperative educational game to increase physical activity in elementary schools. *Smart Health* 19:100163. 10.1016/j.smhl.2020.100163

[B46] Karagiannidis Y. Barkoukis V. Gourgoulis V. Kosta G. Antoniou P. (2015). The role of motivation and metacognition on the development of cognitive and affective responses in physical education lessons: a self-determination approach. *Motricidade* 11 135–150.

[B47] Kemmis S. McTaggart R. Nixon R. (2013). *The Action Research Planner: Doing Critical Participatory Action Research*. Berlin: Springer Science & Business Media.

[B48] Kennedy S. G. Sanders T. Estabrooks P. A. Smith J. J. Lonsdale C. Foster C. (2021). Implementation at-scale of school-based physical activity interventions: a systematic review utilizing the RE-AIM framework. *Obesity Rev.* 22:e13184. 10.1111/obr.13184 33527738

[B49] Kim J. Castelli D. M. (2021). Effects of gamification on behavioral change in education: a meta-analysis. *Int. J. Environ. Res. Public Health* 18:3550. 10.3390/ijerph18073550 33805530 PMC8037535

[B50] Kok G. Gottlieb N. H. Peters G.-J. Y. Mullen P. D. Parcel G. S. Ruiter R. A. C. (2016). A taxonomy of behaviour change methods: an intervention mapping approach. *Health Psychol. Rev.* 10 297–312. 10.1080/17437199.2015.1077155 26262912 PMC4975080

[B51] Kok G. Peters L. W. H. Ruiter R. A. C. Kok G. Peters L. W. H. Ruiter R. A. C. (2017). Planning theory- and evidence-based behavior change interventions: a conceptual review of the intervention mapping protocol. *Psicol.: Reflex. Crít.* 30:19. 10.1186/s41155-017-0072-x 32026109 PMC6975763

[B52] Langford R. Bonell C. P. Jones H. E. Pouliou T. Murphy S. M. Waters E. (2014). The WHO health promoting school framework for improving the health and well-being of students and their academic achievement. *Cochrane Database Syst. Rev.* 4:CD008958. 10.1002/14651858.CD008958.pub2 24737131 PMC11214127

[B53] Lonsdale C. Rosenkranz R. R. Peralta L. R. Bennie A. Fahey P. Lubans D. R. (2013). A systematic review and meta-analysis of interventions designed to increase moderate-to-vigorous physical activity in school physical education lessons. *Preven. Med.* 56 152–161. 10.1016/j.ypmed.2012.12.004 23246641

[B54] Love R. Adams J. Sluijs E. M. F. (2019). Are school-based physical activity interventions effective and equitable? A meta-analysis of cluster randomized controlled trials with accelerometer-assessed activity. *Obesity Rev.* 20 859–870. 10.1111/obr.12823 30628172 PMC6563481

[B55] Mannocci A. D’Egidio V. Backhaus I. Federici A. Sinopoli A. Ramirez Varela A. (2020). Are there effective interventions to increase physical activity in children and young people? An umbrella review. *Int. J. Environ. Res. Public Health* 17:3528. 10.3390/ijerph17103528 32443505 PMC7277151

[B56] Molnar B. E. Gortmaker S. L. Bull F. C. Buka S. L. (2004). Unsafe to play? Neighborhood disorder and lack of safety predict reduced physical activity among urban children and adolescents. *Am. J. Health Promotion* 18 378–386. 10.4278/0890-1171-18.5.378 15163139

[B57] Murillo Pardo B. García Bengoechea E. Generelo Lanaspa E. Bush P. L. Zaragoza Casterad J. Julián Clemente J. A. (2013). Promising school-based strategies and intervention guidelines to increase physical activity of adolescents. *Health Educ. Res.* 28 523–538. 10.1093/her/cyt040 23515117

[B58] Nader P. A. Hilberg E. Schuna J. M. John D. H. Gunter K. B. (2018). Teacher-level factors, classroom physical activity opportunities, and children’s physical activity levels. *J. Phys. Act. Health* 15, 637–643. 10.1123/jpah.2017-0218 29584527

[B59] Ntoumanis N. (2001). Empirical links between achievement goal theory and self-determination theory in sport. *J. Sports Sci.* 19 397–409. 10.1080/026404101300149357 11411776

[B60] Office of Disease Prevention and Health Promotion (ODPHP) (2019). *Healthy People 2030 Framework. U.S. Department of Health and Human Service.* Avaialble online at: https://www.healthypeople.gov/2020/topics-objectives/topic/maternal-infant-and-child-health/objectives (accessed November 30, 2020).

[B61] Ogden C. L. Carroll M. D. Fryar C. D. Flegal K. M. (2015). Prevalence of obesity among adults and youth: United States, 2011–2014. *NCHS Data Brief* 219, 1–8. 26633046

[B62] Parker M. Templin T. Setiawan C. (2012). What has been learned from school-university partnerships. *J. Phys. Educ. Recreat. Dance* 83 32–35. 10.1080/07303084.2012.10598845

[B63] Pate R. R. O’Neill J. R. (2008). Summary of the american heart association scientific statement: promoting physical activity in children and youth: a leadership role for schools. *J. Cardiovasc. Nurs.* 23, 44–49. 10.1097/01.JCN.0000305056.96247.bb 18158507

[B64] Patton K. (2012). Introduction. *J. Phys. Educ., Recreat. Dance* 83 13–14. 10.1080/07303084.2012.10598840

[B65] Pelletier L. G. Tuson K. M. Fortier M. S. Vallerand R. J. Briére N. M. Blais M. R. (1995). Toward a new measure of intrinsic motivation, extrinsic motivation, and amotivation in sports: the Sport Motivation Scale (SMS). *J. Sport Exercise Psychol.* 17 35–53. 10.1123/jsep.17.1.35

[B66] Pfledderer C. D. Burns R. D. Byun W. Carson R. L. Welk G. J. Brusseau T. A. (2021). School-based physical activity interventions in rural and urban/suburban communities: a systematic review and meta-analysis. *Obesity Rev.* e13265. 10.1111/obr.13265 33938109

[B67] Phelps A. Jung Y. Castelli D. (2019). “Multicomponent optimization strategy and CSPAP implementation,” in *Comprehensive School Physical Activity Program: Putting Research into Evidence-Based Practice*, eds Carson R. Webster C. A. (Champaign, IL: Human Kinetics Publishers), 157–170.

[B68] Piercy K. L. Troiano R. P. Ballard R. M. Carlson S. A. Fulton J. E. Galuska D. A. (2018). The physical activity guidelines for Americans. *JAMA* 320:2020. 10.1001/jama.2018.14854 30418471 PMC9582631

[B69] Ploeg K. A. V. McGavock J. Maximova K. Veugelers P. J. (2014). School-based health promotion and physical activity during and after school hours. *Pediatrics* 133 e371–e378. 10.1542/peds.2013-2383 24420806

[B70] Pulling Kuhn A. Stoepker P. Dauenhauer B. Carson R. L. (2021). A systematic review of multi-component Comprehensive School Physical Activity Program (CSPAP) interventions. *Am. J. Health Promotion [Online ahead of print]* 08901171211013281. 10.1177/08901171211013281 33955278

[B71] Ryan R. M. Deci E. L. (2000). Self-determination theory and the facilitation of intrinsic motivation, social development, and well-being. *Am. Psychol.* 55 68–78. 10.1037/0003-066X.55.1.68 11392867

[B72] Sergis S. Sampson D. G. Pelliccione L. (2018). Investigating the impact of Flipped Classroom on students’ learning experiences: a self-determination theory approach. *Comput. Hum. Behav.* 78 368–378. 10.1016/j.chb.2017.08.011

[B73] Sevil-Serrano J. Aibar A. Abós A. Generelo E. García-González L. (2020). Improving motivation for physical activity and physical education through a school-based intervention. *J. Exp. Educ.* 1–21. 10.1080/00220973.2020.1764466

[B74] Stratton G. Mullan E. (2005). The effect of multicolor playground markings on children’s physical activity level during recess. *Prev. Med.* 41, 828–833. 10.1016/j.ypmed.2005.07.009 16137756

[B75] Teixeira P. J. Marques M. M. Silva M. N. Brunet J. Duda J. L. Haerens L. (2020). A classification of motivation and behavior change techniques used in self-determination theory-based interventions in health contexts. *Motivation Sci.* 6 438–455. 10.1037/mot0000172

[B76] Tran I. Clark B. R. Racette S. B. (2013). Physical activity during recess outdoors and indoors among urban public school students, St. Louis, Missouri, 2010-2011. *Prev. Chronic. Dis.* 10:E196. 10.5888/pcd10.130135 24262028 PMC3839587

[B77] Trost S. G. Pate R. R. Ward D. S. Saunders R. Riner W. (1999). Correlates of objectively measured physical activity in preadolescent youth. *Am. J. Prev. Med.* 17, 120–126. 10.1016/S0749-3797(99)00056-2 10490054

[B78] Vasconcellos D. Parker P. D. Hilland T. Cinelli R. Owen K. B. Kapsal N. (2020). Self-determination theory applied to physical education: a systematic review and meta-analysis. *J. Educ. Psychol.* 112 1444–1469. 10.1037/edu0000420

[B79] Wallace H. S. (2019). Community coalitions for change and the policy, systems, and environment model: a community-based participatory approach to addressing obesity in rural tennessee. *Prevent. Chronic Dis.* 16:180678. 10.5888/pcd16.180678 31489837 PMC6745929

[B80] Wilson D. K. Van Horn M. L. Kitzman-Ulrich H. Saunders R. Pate R. Lawman H. G. (2011). Results of the “Active by Choice Today” (ACT) randomized trial for increasing physical activity in low-income and minority adolescents. *Health Psychol.* 30 463–471. 10.1037/a0023390 21534677 PMC3417297

[B81] World Health Organization (WHO) (2019). *Global Action Plan on Physical Activity 2018-2030: More Active People for a Healthier World*. NC SA 3.0. South Perth: IGO.

